# Antimicrobial Activity of Red Alga Flour (*Gelidium* sp.) and Its Effect on Quality Retention of *Scomber scombrus* during Refrigerated Storage

**DOI:** 10.3390/foods11070904

**Published:** 2022-03-22

**Authors:** José M. Miranda, Marcos Trigo, Jorge Barros-Velázquez, Santiago P. Aubourg

**Affiliations:** 1 Department of Analytical Chemistry, Nutrition and Food Science, School of Veterinary Sciences, University of Santiago de Compostela, 27002 Lugo, Spain; josemanuel.miranda@usc.es (J.M.M.); jorge.barros@usc.es (J.B.-V.); 2 Department of Food Science and Technology, Marine Research Institute (CSIC), 36208 Vigo, Spain; mtrigo@iim.csic.es

**Keywords:** *Gelidium* sp., flour, antimicrobial activity, in vitro assay, aqueous extract, mackerel, chilled storage, quality enhancement

## Abstract

This study analyzed the antimicrobial effect of aqueous extracts of flour obtained from red alga (*Gelidium* sp.) both in vitro, against most common food pathogenic and spoilage bacteria, and in a food model system during the chilled storage of Atlantic mackerel (*Scomber scombrus*). Results of in vitro assays allowed the conclusion that the aqueous flour extracts have antimicrobial activity against Gram-negative bacteria such as *Enterobacteriaceae* (*Escherichia coli*, *Enterobacter aerogenes*, and *Klebsiella pneumoniae*) and proteobacteria (*Vibrio alginolyticus*), and against Gram-positive bacteria such as *Bacillus cereus* and *B. subtilis*. In the food model study, different concentrations of the flour extract were present in the icing medium, microbial and chemical analyses being carried out in fish muscle at different storage times. An inhibitory effect (*p* < 0.05) on microbial growth (aerobes, psychrotrophs, *Enterobacteriaceae*, and proteolytic and lipolytic bacteria) and on chemical quality indices (pH, total volatile amines, and trimethylamine) was concluded. This effect was more pronounced when the flour extract concentration in the ice increased and at advanced storage times. This study provides a first approach to the beneficial use of flour of the alga *Gelidium* as a new preserving strategy for chilled fish.

## 1. Introduction

Fresh fish deteriorate rapidly after death due to a variety of degradation mechanisms [1,2]. Among such mechanisms, microbial activity represents a major concern as a consequence of certain characteristics of marine species such as their high content of water and non-protein nitrogen compounds, as well as their low proportion of connective tissue and their poikilothermic nature [3]. Therefore, the development of innovative chilling, packaging and distribution technologies focused on inhibiting microbial activity for refrigerated fish species has increased notably in the last decades [4,5]. Among them, the replacement of synthetic preservatives with those obtained from natural resources has received increasing attention [6,7].

For centuries, marine algae have been part of the human diet in different Asian countries. They have been commonly classified into three groups based on their pigmentation, i.e., brown (*Phaeophyceae*), red (*Rhodophyceae*), and green (*Chlorophyceae*) algae. Marine algae are known for their richness in polysaccharides, minerals, ω-3 and ω-6 polyunsaturated fatty acids, vitamins, and a wide range of bioactive substances exhibiting preservative properties [8,9,10]. Interestingly, marine algae have been considered by the European Council [11] as a food or food ingredient, so their use would not pose a health problem for consumers. In this sense, the use of different types of macroalga extracts for microbial inhibition in fish during refrigerated storage has been evaluated. Accordingly, different strategies have been developed, i.e., previous dipping in an alga extract [12], including an alga extract in the icing system [13], or including a lyophilized alga in the packaging medium [14].

Red macroalgae are especially abundant in marine habitats and are consumed raw, in salads, soups, meal, and as condiments. In addition to their remarkable level of constituents exhibiting high nutritional value [15], this alga group has been shown to be a rich source of preservative compounds, these including antibacterial [16,17], antioxidant [18,19], antifungal [20], and pharmaceutical [21] properties. Most red macroalgae are primarily known for their industrial use for extracting phycocolloids (agar, algin, furcellaran, and carrageenan) [22,23,24], being used to enhance the physical properties of edible films in novel food packaging applications [25,26]. Notably, recent studies have concluded a microbial inhibition effect when red alga extracts are included in the storage strategies of refrigerated fish [27,28,29].

Accordingly, the present study was aimed to evaluate, to our knowledge for the first time, the antimicrobial effect of flour obtained from the red alga *Gelidium* sp., an organism commonly employed for animal feeding but poorly studied and underutilized in food applications. Accordingly, the in vitro activity of a *Gelidium* flour extract was evaluated against most common pathogenic and spoilage bacteria. In addition, such extract was evaluated for the refrigerated storage of Atlantic mackerel (*Scomber scombrus*) by the inclusion in the icing medium. Microbial and chemical analyses were carried out in fish muscle at different storage times to assess the inhibitory effect on microbial development.

## 2. Materials and Methods

### 2.1. Alga Flour

Commercial flour obtained from *Gelidium* sp. was provided by Industrias Roko S. A. (Llanera, Asturias, Spain). According to information provided by the company, the alga flour exhibited the following proximate composition (%): 12.2 (moisture), 31.5 (protein), 0.2 (lipids), 14.3 (ash), and 28.5 (total fiber).

### 2.2. In Vitro Analysis of Microbial Activity

The antimicrobial activity of *Gelidium* flour extracts against selected pathogenic and spoilage bacteria listed in Table 1 was evaluated by measuring the diameter of the inhibition zone by means of the disk diffusion method. Thus, pathogenic and spoilage bacteria were cultured at 30 °C in Mueller–Hinton broth (Oxoid Ltd., London, UK), except for *Vibrio parahaemolyticus* and *V. alginolyticus*, which were incubated in BHI broth (Oxoid) and Marine broth (Merck, Darmstadt, Germany), respectively. Once grown, the cultures were adjusted to a 0.5 McFarland value, this corresponding to a microbial concentration of 1.5 × 10^8^ CFU·mL^−1^. Afterwards, sterile swabs were used for seeding each bacterial strain on Mueller–Hinton agar (Oxoid) plates, except for *V. parahaemolyticus* and *V. alginolyticus*, which were seeded on BHI agar (Oxoid) and Marine agar (Merck) plates, respectively.

Then, 6 mm sterile disks were placed in the plates and inoculated with either 10, 20, or 25 μL of the alga flour extract. The alga flour extract was prepared as follows. Briefly, a mixture of 1 g of alga flour and 5 mL of sterile distilled water was prepared, extraction being extended to 24 h under agitation in the absence of light. Afterwards, the extracts were centrifuged for 10 min at 1500 rpm, the supernatants being recovered and stored under refrigeration until analysis. A disk containing 30 μg of chloramphenicol was used as a positive control. All plates were incubated for 24 h at 30 °C. Finally, the presence of inhibition zones surrounding the disks was investigated. When inhibition zones were detected, the diameters were measured (in mm) to estimate the intensity of bacterial inhibition.

The minimum inhibitory concentrations (MICs) of the *Gelidium* flour extract against selected pathogenic and spoilage bacteria were investigated in the range 0.3125–100 mg·mL^−1^ *w*·*v*^−1^. Thus, serial dilutions of the alga extract were prepared and mixed with equal volumes of bacterial suspensions. For each bacterial species, the MIC was determined as the lowest extract concentration exhibiting antimicrobial activity after 24 h of incubation at 30 °C.

### 2.3. Study of Microbial Activity Inhibition in Chilled Fish

#### 2.3.1. Preparation of Icing Media Including the Alga Flour Extract

A mixture of alga flour (26 g) and distilled water (400 mL) was stirred for 30 s, sonicated for 30 s, and centrifuged at 3500× *g* for 30 min at 4 °C. After recovering of the supernatant, the extraction process was carried out three more times. Finally, all four supernatants were pooled together and made up to 2 L by employing distilled water.

Three concentrations of the alga flour extract (AFE) were taken into account for preparing the different kinds of icing media. For preparing the least concentrated ice, 150 mL of the above-mentioned extract (i.e., corresponding to 2 g of alga flour) were carried to 6 L with distilled water. The resulting solution was placed in a polyethylene bag, stored frozen at −18 °C and later considered as icing medium (low AFE content; AFE-1). To prepare the two other flour icing media, 450 and 1350 mL of the flour extract (i.e., corresponding to 6 and 18 g of alga flour, respectively) were taken and carried, respectively, to 6 L with distilled water. The resulting solutions were also placed in polyethylene bags, stored frozen at −18 °C and later used as icing media (medium and high AFE content; AFE-2 and AFE-3, respectively). Control ice (CT) was prepared starting from 6 L of distilled water that were kept frozen in the same way as the above-mentioned solutions including the AFE. Before use as icing media for fish chilling, control and AFE-containing ices were ground to obtain common ice flakes.

Current experimental conditions (AFE content in ice) employed in this study were based on preliminary trials developed in our laboratory. Under the present experimental conditions, 1350 mL of flour extract in a 6 L solution (AFE-3 batch) would correspond to the highest concentration possible that does not modify the sensory acceptance of fish (i.e., flesh color, odor, or flavor). In order to analyze the effect of the alga flour content, two smaller volumes of the AFE extract (150 and 450 mL, AFE-1 and AFE-2 batches, respectively) were considered in the present research.

#### 2.3.2. Raw Fish, Chilled Storage, and Sampling Procedure

Fresh Atlantic mackerel (*Scomber scombrus*) (81 specimens) were caught off the Galician Atlantic coast (Vigo, Northwestern Spain) and carried in ice to the laboratory (10 h). The weight and length of the fish specimens were in the 220–280 g and 29–33 cm ranges, respectively.

Once in the laboratory, nine individual fish specimens were taken and analyzed as initial material (day 0). Such fish specimens were divided into three different groups (three specimens in each group) that were analyzed independently (*n* = 3). The remaining fish specimens were distributed into four groups (18 specimens in each group), that were placed in different boxes and directly surrounded by the different above-mentioned kinds of ice (CT, AFE-1, AFE-2, and AFE-3 batches, respectively). All fish batches were placed inside a refrigerated room (4 °C). During storage, boxes allowing the drainage of melted ice were used in all batches, ice being renewed when necessary to maintain a 1:1 fish:ice ratio. Fish specimens were taken for analysis on days 4 and 11. At each sampling time, nine specimens were taken from each batch for microbial and chemical analyses and divided into three groups (three specimens in each group) that were analyzed independently (*n* = 3).

#### 2.3.3. Microbial Analyses

These were performed in samples of 10 g which were dissected from fish muscle and were mixed with 90 mL of 0.1% peptone water (Merck, Darmstadt, Germany). The mixtures were homogenized in sterilized stomacher bags (AES, Combourg, France) as previously reported [30,31]. All extracts were diluted in 0.1% peptone water.

Aerobic mesophiles were investigated in plate count agar (PCA) (Oxoid) after incubation for 48 h at 30 °C. Psychrotrophs were also investigated in PCA after 7 days of incubation at 7 °C. The investigation of *Enterobacteriaceae* was carried out in Violet Red Bile Agar (VRBA) (Merck) after 24 h of incubation at 37 °C. Bacteria exhibiting either a lipolytic or proteolytic phenotype were investigated in tributyrin agar or casein agar, respectively, after 48 h of incubation at 30 °C, as previously reported [32]. All microbial analyses were performed in triplicate.

#### 2.3.4. Chemical Analyses

The evolution of pH values in mackerel white muscle with storage time was determined using a 6 mm-diameter insertion electrode (Crison, Barcelona, Spain).

Assessment of total volatile base-nitrogen (TVB-N) values was carried out as reported by Antonacopoulos [33], with some modifications. For it, 10 g of fish white muscle were extracted with 30 mL of 60 g·L^−1^ perchloric acid in water and carried to 50 mL. An aliquot of the acid extracts was rendered alkaline to pH 13 with 200 g·L^−1^ aqueous NaOH and then steam-distilled. The TVB-N value was assessed by titration of the distillate with a 10 mM HCl solution. Results were calculated as mg TVB-N·kg^−1^ fish muscle.

Trimethylamine-nitrogen (TMA-N) values were assessed by employing the picrate spectrophotometric method (410 nm; Beckman Coulter DU 640 spectrophotometer, Beckman Coulter Inc., Brea, CA, USA), according to the Tozawa et al. [34] procedure. For it, a 5% trichloroacetic acid extract of fish white muscle (10 g in 25 mL) was prepared. Results were calculated as mg TMA-N·kg^−1^ fish muscle.

#### 2.3.5. Statistical Analysis

Microbiological and chemical data obtained from all analyses were subjected to the ANOVA method in order to explore differences resulting from the effect of the icing system employed during chilling storage and the chilling time employed. For it, the comparison of means was carried out by employing the least-squares difference (LSD) method. In all cases, the statistical study was carried out using PASW Statistics 18 software for Windows (SPSS Inc., Chicago, IL, USA); a significant confidence interval at the 95% level (*p* < 0.05) was considered in all cases.

## 3. Results

### 3.1. Study on In Vitro Microbial Activity

Disk diffusion is a fast method to measure antimicrobial activity. The results obtained in this study for the *Gelidium* flour extract are compiled in Table 2. Remarkably, the highest volume of alga extract tested (25 µL) resulted in the successful inhibition of most bacterial species tested, including both Gram-positives and Gram-negatives. In contrast, *Ps. fluorescens* and *Ps. putida* exhibited resistance to the antimicrobial components of the alga extract. Only *B. subtilis* and *S. enterica* were inhibited by the lowest volume of the alga extract tested (10 µL), while 7 of the 13 species tested were inhibited by 15 µL of the alga extract. The highest level of inhibition was observed both for Gram-negatives such as *Enterobacteriaceae* (*E. coli*, *E. aerogenes*, and *K. pneumoniae*) and proteobacteria (*V. alginolyticus*), and for Gram-positives such as *B. cereus* and *B. subtilis*.

Table 3 compiles the results of the MICs determined for 9 of the 13 bacterial species tested. Interestingly, the lowest MICs were determined for *B. cereus* and *B. subtilis*, which were the most sensitive to the alga flour extract. The remaining bacterial species, either Gram-negatives or Gram-positives, exhibited moderate sensitivity to low extract concentrations, the MICs being in the range 12.5–50 mg·mL^−1^.

### 3.2. Study on Microbial Activity Inhibition during Fish Chilling

#### 3.2.1. Microbial Parameters

**Figure 1:**
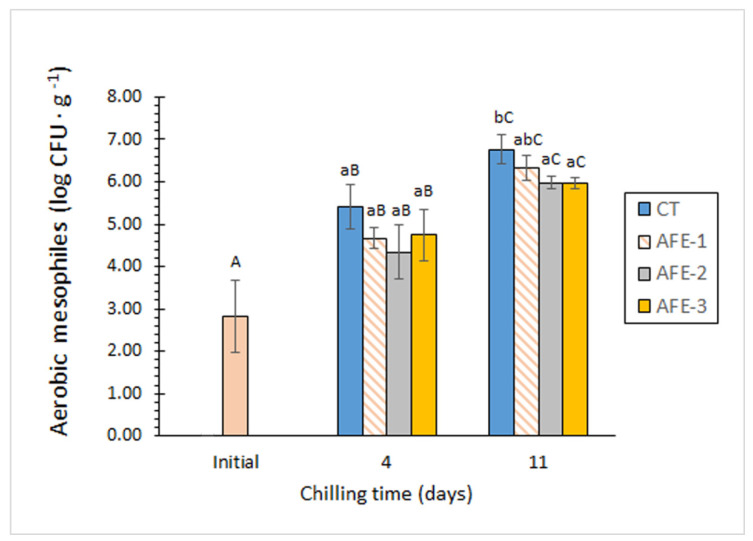
Aerobic mesophiles (log CFU·g^−1^ muscle) in chilled mackerel subjected to different icing conditions. Mean values of three replicates (*n* = 3); standard deviations are expressed by bars. Mean values accompanied by different lowercase letters (a, b) denote significant differences (*p* < 0.05) as a result of icing conditions; mean values accompanied by different capital letters (A–C) denote significant differences (*p* < 0.05) as a result of storage time. Icing conditions: CT (control; ice prepared without alga flour extract); AFE-1, AFE-2, and AFE-3 correspond to low, medium, and high concentrations of alga flour extracts, respectively.

Figure 1 shows the effect of *Gelidium* flour extracts on the evolution and growth of aerobic mesophiles in mackerel muscle during 11 days of refrigerated storage. All three AFE batches exhibited lower average microbial counts compared to the CT batch at both sampling times. Remarkably, such differences were significant (*p* < 0.05) for AFE-2 and AFE-3 batches at day 11 compared to the CT batch. It should also be noted that neither AFE-2 and AFE-3 batches reached mesophile counts above 6 log CFU·g^−1^, while AFE-1 and CT batches surpassed that level at day 11. Accordingly, the addition of *Gelidium* flour extract to the icing medium provided a slight protective effect with respect to aerobe growth in mackerel muscle. A similar result was observed for psychrotrophs (Table 4). Thus, both AFE-2 and AFE-3 batches provided significantly (*p* < 0.05) better control of this microbial group compared to the CT batch at day 11. In this sense, both AFE-2 and AFE-3 batches, with the highest concentrations of alga extracts, provided microbial concentrations below 7 log CFU·g^−1^, while the values determined for AFE-1 and CT batches were above that level. As in the case of aerobes, the addition of *Gelidium* flour extract to the icing medium implied a slightly better control of psychrotrophs compared to the control batch.

With respect to the growth of *Enterobacteriaceae* in mackerel muscle, an inhibitory effect was also observed at day 11 as a result of adding *Gelidium* flour extract to the icing medium (Table 4). Such an effect was found to be significant (*p* < 0.05) when comparing AFE-3 and CT batches at day 11 and implied a microbial reduction of 1.11 log units (Table 4). Thus, and as in the case of aerobes and psychrotrophs, a slight antimicrobial effect derived from the presence of the alga extract in the icing medium of mackerel could be inferred.

The investigation of bacteria exhibiting a lipolytic phenotype was also performed in mackerel muscle. Such bacteria may enhance lipid hydrolysis, a mechanism with a remarkable negative effect on mackerel quality during chilled storage. As can be observed in Table 4, all three AFE batches exhibited lower average counts of this microbial group compared with the CT batch at both sampling times. However, a significant (*p* < 0.05) difference occurred only at day 4 and was not reached at advanced storage times (day 11). The greatest reduction of lipolytic bacteria counts (1.07 units) was observed when comparing AFE-2 and CT batches at day 4.

**Figure 2:**
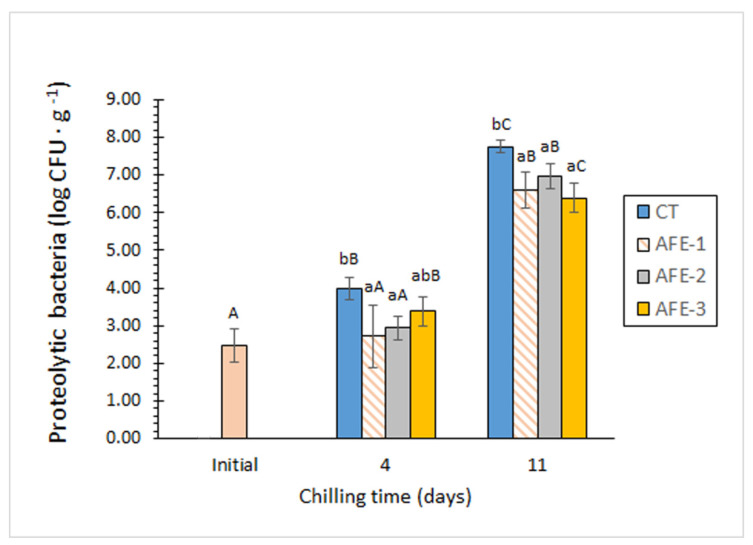
Proteolytic bacteria (log CFU·g^−1^ muscle) in chilled mackerel subjected to different icing conditions. Mean values of three replicates (*n* = 3); standard deviations are expressed by bars. Mean values accompanied by different lowercase letters (a, b) denote significant differences (*p* < 0.05) as a result of icing conditions; mean values accompanied by different capital letters (A–C) denote significant differences (*p* < 0.05) as a result of storage time. Icing conditions (CT, AFE-1, AFE-2, and AFE-3) as expressed in Figure 1.

Proteolytic bacteria are also specific spoilage bacteria that contribute to protein breakdown and subsequent quality loss of mackerel muscle during chilled storage. As can be observed in Figure 2, the addition of *Gelidium* flour extract to the icing medium provided better control of this microbial group compared with the CT batch. Such a result indicates a protective effect of the alga extract against the development and growth of bacteria exhibiting a proteolytic phenotype in mackerel muscle. Thus, Figure 2 displays how all three AFE batches exhibited lower average counts of proteolytic bacteria at both sampling times compared with the CT batch. Moreover, such differences were found to be significant (*p* < 0.05) in all cases except for the AFE-3 batch at day 4, reaching microbial reductions of 1.26 log units (AFE-1 batch, day 4) and 1.37 log units (AFE-3 batch, day 11) compared with the CT batch counterparts. From these results, the inhibitory effect of *Gelidium* flour extract on the growth of bacteria exhibiting a proteolytic phenotype in mackerel muscle during chilled storage can be concluded, thus implying a remarkable protective effect of such fish species against protein hydrolysis events of microbial origin.

#### 3.2.2. Chemical Parameters

Chemical assessment of microbial activity in chilled mackerel muscle was carried out by means of pH, TVB-N, and TMA-N analyses.

The pH assessment showed a progressive increase of the average value with storage time in all batches (Table 5). Differences among batches were scarce, these showing a lower (*p* < 0.05) value in fish specimens corresponding to the AFE-1 batch compared to their CT counterparts. Remarkably, no differences (*p* > 0.05) were detected among batches including alga flour in the icing medium, so that no effect (*p* > 0.05) of the flour concentration tested could be concluded. Increases in the pH value of fish muscle during storage have been reported to indicate the accumulation of alkaline compounds, such as ammonia, TMA, and other nitrogen-containing compounds, which are mainly derived from microbial spoilage [1,3]. However, the pH values determined in this study did not show remarkable differences as a result of the presence of flour in the different icing media.

The formation of total volatile amines was not significant (*p* > 0.05) after 4 days of chilled storage in any of the fish batches considered. However, a general increase of the average amine content was detected in all batches at day 11, this increase being significant (*p* < 0.05) in all fish batches, except for AFE-3. Notably, a lower value (*p* < 0.05) of this deteriorative index was observed in mackerel specimens corresponding to the AFE-3 batch, as compared to the CT batch. Consequently, an inhibitory effect of the active ice including alga flour extract on total volatile amine formation was concluded.

Marked formation of TMA (*p* < 0.05) was detected in all batches during the chilled storage (Table 5). Comparison among batches showed lower (*p* < 0.05) average TMA levels when the presence of alga flour in the icing medium increased. At both chilling times (i.e., 4 and 11 days), all alga-treated fish specimens exhibited lower (*p* < 0.05) TMA-N values than fish specimens belonging to the CT batch. Accordingly, an inhibitory effect on TMA formation is concluded for the alga-flour icing media. Remarkably, no significant differences (*p* > 0.05) were detected among the three batches including alga flour.

Both TVB-N and TMA-N values are considered closely related indicators of microbial activity [2,5]. Thus, proteins in seafood are known to be decomposed by endogenous enzymes and spoilage bacteria, resulting in the formation of nitrogen-containing volatile amines. Furthermore, TMA is reported to be produced during the chilled storage of fish as a result of the breakdown of trimethylamine oxide (TMAO) by microbial TMAO reductase. Interestingly, values determined for TVB-N and TMA-N were in agreement with the results obtained in the microbial analyses, thus showing the microbial inhibitory effect derived from the presence of the alga flour extract in the icing system.

## 4. Discussion

The flour obtained from red *Gelidium* sp. in this study exhibited antimicrobial activity according to in vitro analyses, a result that was also observed during the chilled storage of mackerel. Previous research concerning the antimicrobial effect of red alga constituents in both in vitro and in vivo studies has already been published. In them, different kinds of molecules have been found to be responsible for such a preservative effect, according to the kind of extracting medium employed (i.e., water, ethanol, methanol, ethyl acetate, etc.). Analysis of molecules responsible for the inhibitory effect was not carried out in the present study. However, and on the basis that a water extract of red alga flour was currently used, antimicrobial activity found can be explained as a result of the presence of hydrophilic constituents from red alga flour. Notably, previous research has shown the antimicrobial properties of aqueous extracts of red alga and of hydrophilic constituents obtained from red alga.

Thus, El-Baroty et al. [35] reported that the glycolipids obtained from two red algae (*Laurencia papillosa* and *Galaxaura cylindrica*) exhibited antimicrobial properties (in vitro assays) due to their high content of monosaccharide compounds (mannuronic acid, galactose, and rhamnose). Moreover, Zeid et al. [36] tested cold and hot water extracts from the red alga *Pterocladia capillacea* and described remarkable antimicrobial activity in vitro; thus, while the cold water extract was rich in glucuronic acid, arabinose, and glucose, the hot extract was found to be rich in glucuronic acid and fructose. Sulfated polysaccharide isolated from *Gracilaria corticata* has shown good antibacterial activity against human pathogens (i.e., *Salmonella* Typhi, *Salmonella paratyphi*, *Staphylococcus aureus*, *Vibrio cholerae*, and *Klebsiella oxytoca*) [22]. Cui et al. [21] also reported that *Gelidium pacificum* sulfated polysaccharide exhibited beneficial effects on mice by promoting the recovery of their gut microbiota and mucosal barrier function.

The antimicrobial activity of aqueous and ethanolic extracts of the red alga *Gelidium pusillum* was recently studied by Agarwal et al. [19]; thus, in vitro analysis showed an inhibitory effect on the marine pathogen *Aeromonas caviae*, whereas in vivo assays showed that freshwater Giant prawns (*Macrobrachium rosenbergii*) survived even after 3 weeks whereas untreated prawns showed 100% mortality within 120 h. Also recently, Ortiz-Viedma et al. [17] investigated the antimicrobial activity of ethanol and aqueous extracts of the red algae *Gracilaria chilensis*, *Gelidium chilense*, *Iridaea larga*, *Gigartina chamissoi*, *Gigartina skottsbergii*, and *Gigartina radula*; according to in vitro assays, all these alga species showed antibacterial activity against *Salmonella enteritidis*, *B. cereus*, and *E. coli*.

With respect to microbial development in seafood during storage, previous research has already proved an inhibitory effect of aqueous extracts of red algae. Thus, Lim et al. [27] analyzed the antimicrobial properties of a *Gelidium corneum*–whey protein isolate film containing grapefruit seed extract; an antimicrobial effect was proved on fish paste inoculated with *E. coli* O157:H7, *Listeria monocytogenes*, and *Salmonella typhimurium* during 12 days of storage at 4 °C, this leading to the treated product having an extended shelf-life. Afterwards, Barbosa et al. [28] incorporated an ethanolic and aqueous extract of the red alga *Gracilaria gracilis* in the icing medium to be applied during 9 days of chilled storage of hake (*Merluccius merluccius*); as a result, an inhibitory effect on TMA formation was concluded. An inhibited microbial activity was also implied in chilled Black Tiger shrimp (*Penaeus monodon*) when specimens were previously soaked in 5% ethanolic extracts obtained from red seaweed (*Hypnea musciformis* and *Acanthophora muscoides*) [29]; lower biogenic amine formation and an enhanced shelf-life were observed.

The inclusion of aqueous extracts of non-red macroalgae in the ice system used for fish chilling has also previously been tested as a preservation strategy. Thus, an ethanolic and aqueous extract of *Cystoseira compressa* was included in the icing system employed during 11 days of chilled storage of a medium-fat fish species (horse mackerel, *Trachurus trachurus*) [13]; inhibition of microbial activity was detected as determined by microbial parameters (detection of aerobe, psychrotroph, proteolytic, lipolytic, and *Enterobacteriaceae* counts) and chemical indices related to microbial activity (TMA and pH levels). Recently, aqueous and ethanolic extracts of *Bifurcaria bifurcata* were also incorporated in the icing system employed during storage of hake (*Merluccius merluccius*) [37]; substantial inhibition of microbial activity was observed (as determined by counts of psychrotrophs and lipolytic bacteria) resulting from both kinds of extracts during 13 days of storage.

## 5. Conclusions

Red macroalgae constitute an important source of beneficial constituents and biomolecules. This study analyzed the antimicrobial effect of flour obtained from red alga *Gelidium* sp. Results of in vitro assays indicated the inhibition of most bacterial species tested, these including both Gram-positives and Gram-negatives, by aqueous extracts prepared from alga flour. Concerning the fish refrigeration study, an inhibitory effect (*p* < 0.05) on microbial growth (aerobic mesophiles, psychrotrophs, *Enterobacteriaceae*, proteolytic bacteria and lipolytic bacteria) and on chemical quality indices related to microbial development (pH, total volatile amines, and TMA) was also concluded. This effect was found to be more pronounced both when the flour extract concentration in the icing medium was increased and at advanced times of mackerel storage. On the basis that an aqueous extract of alga flour was employed in the present study, hydrophilic compounds present in the alga flour are considered as potential active compounds responsible for this preservative behavior.

This study can be considered a novel and beneficial strategy to employ flour obtained from red macroalgae (i.e., *Gelidium* sp.). The present study agrees with current global interests in food technology in the search for new strategies including preservative compounds obtained from natural sources. According to the simplicity of the methodology carried out and the abundancy of *Gelidium* sp., further research is envisaged to optimize extraction of the preservative compounds and to analyze the molecules involved in this preservative action.

## Figures and Tables

**Table 1:** Pathogenic and spoilage bacteria selected for the microbial inhibition assay.

*Salmonella enterica* CECT 4396
*Pseudomonas fluorescens* ATCC 17397
*Pseudomonas putida* ATCC 12633
*Escherichia coli* CECT 4387
*Bacillus subtilis* ATCC 6051
*Bacillus cereus* CECT 148
*Bacillus licheniformis* ATCC 27811
*Vibrio alginolyticus* ATCC 17749
*Vibrio parahaemolyticus* ATCC 17802
*Proteus mirabilis* ATCC 29906
*Enterobacter aerogenes* ATCC 13048
*Listeria monocytogenes* CECT 4032
*Klebsiella pneumoniae* ATCC 9997

**Table 2:** Antimicrobial activity of *Gelidium* flour extract on pathogenic and spoilage bacteria.

	Diameter of Inhibition Zone (mm)		
	Volume of Alga Extract		
Bacterial Species	10 µL	20 µL	25 µL
*Salmonella enterica* CECT 4396	10	11	12
*Pseudomonas fluorescens* ATCC 17397	ND *	ND	ND
*Pseudomonas putida* ATCC 12633	ND	ND	ND
*Escherichia coli* CECT 4387	ND	13	14
*Bacillus subtilis* ATCC 6051	10	12	13
*Bacillus cereus* CECT 148	ND	11	14
*Bacillus licheniformis* ATCC 27811	ND	ND	11
*Vibrio alginolyticus* ATCC 17749	ND	ND	13
*Vibrio parahaemolyticus* ATCC17802	ND	11	12
*Proteus mirabilis* ATCC 29906	ND	ND	11
*Enterobacter aerogenes* ATCC 13048	ND	11	13
*Listeria monocytogenes* CECT 4032	ND	ND	11
*Klebsiella pneumoniae* ATCC 9997	ND	11	13

* ND: not detected.

**Table 3:** Minimum inhibitory concentration of *Gelidium* flour extract on selected bacteria.

Bacterial Species	Breakpoint *
*Salmonella enterica* CECT 4396	12.5 mg·mL^−1^
*Escherichia coli* CECT 4387	50 mg·mL^−1^
*Bacillus subtilis* ATCC 6051	3.125 mg·mL^−1^
*Bacillus cereus* CECT 148	0.625 mg·mL^−1^
*Bacillus licheniformis* ATCC 27811	25 mg·mL^−1^
*Proteus mirabilis* ATCC 29906	50 mg·mL^−1^
*Enterobacter aerogenes* ATCC 13048	25 mg·mL^−1^
*Listeria monocytogenes* CECT 4032	50 mg·mL^−1^
*Klebsiella pneumoniae* ATCC 9997	50 mg·mL^−1^

* Lowest concentration exhibiting antimicrobial activity.

**Table 4:** Microbial development (log CFU·g^−1^ muscle) * in chilled mackerel subjected to different icing conditions **.

Microbial Group	Icing Condition	Chilling Time (Days)		
		0	4	11
Psychrotrophs	CT	2.99 A (0.53)	3.89 aB (0.15)	7.65 bC (0.28)
	AFE-1		3.53 aA (0.66)	7.65 bB (0.37)
	AFE-2		3.58 aA (0.48)	6.29 aB (0.04)
	AFE-3		3.80 aB (0.21)	6.36 aC (0.07)
*Enterobacteriaceae*	CT	1.00 A (0.00)	1.10 aA (0.17)	3.30 bB (0.30)
	AFE-1		1.10 aA (0.17)	2.51 abB (0.57)
	AFE-2		1.10 aA (0.17)	3.11 bB (0.64)
	AFE-3		1.20 aA (0.35)	2.19 aB (0.61)
Lipolytics	CT	2.00 A (0.00)	3.47 bB (0.16)	5.65 aC (0.30)
	AFE-1		2.75 aB (0.12)	5.34 aC (0.44)
	AFE-2		2.40 aA (0.46)	5.28 aB (0.43)
	AFE-3		2.79 aB (0.31)	5.50 aC (0.61)

* Mean values of three replicates (*n* = 3); standard deviations are indicated in brackets. Mean values followed by different lowercase letters (a, b) denote significant differences (*p* < 0.05) as a result of icing condition; mean values followed by different capital letters (A–C) denote significant differences (*p* < 0.05) as a result of storage time. ** Icing conditions (CT, AFE-1, AFE-2, and AFE-3) as expressed in Figure 1.

**Table 5:** Evolution of chemical parameters * related to microbial development in chilled mackerel subjected to different icing conditions **.

Chemical Parameter	Icing Condition	Chilling Time (Days)		
		0	4	11
pH	CT	5.70 A (0.02)	5.89 aB (0.09)	6.14 bC (0.05)
	AFE-1		5.80 aA (0.08)	5.99 aB (0.06)
	AFE-2		5.91 aAB (0.17)	6.04 abB (0.12)
	AFE-3		5.97 aB (0.11)	6.04 abB (0.14)
Total volatile base-nitrogen (TVB-N; mg·kg^−1^ muscle)	CT	257.15 A (5.14)	257.10 aA (23. 50)	315.77 bB (5.62)
	AFE-1		260.66 aA (6.31)	311.23 bC (4.52)
	AFE-2		260.60 aA (6.77)	300.21 abB (21.60)
	AFE-3		269.18 aAB (19.01)	283.48 aB (14.28)
Trimethylamine-nitrogen (TMA-N; mg·kg^−1^ muscle)	CT	0.55 A (0.13)	3.43 bB (0.71)	23.33 bC (0.43)
	AFE-1		1.67 aB (0.48)	13.61 aC (1.73)
	AFE-2		1.41 aB (0.43)	12.95 aC (4.13)
	AFE-3		1.83 aB (0.58)	11.59 aC (3.85)

* Mean values of three replicates (*n* = 3); standard deviations are indicated in brackets. Mean values followed by different lowercase letters (a, b) denote significant differences (*p* < 0.05) as a result of storage temperature; mean values followed by different capital letters (A–C) denote significant differences (*p* < 0.05) as a result of storage time. ** Icing conditions (CT, AFE-1, AFE-2, and AFE-3) as expressed in Figure 1.

## Data Availability

Not applicable.
